# Apoptosis signal-regulating kinase 1 is associated with the effect of claudin-6 in breast cancer

**DOI:** 10.1186/1746-1596-7-111

**Published:** 2012-08-27

**Authors:** Yaxiong Guo, Xiaoming Xu, Zhijing Liu, Ting Zhang, Xiaowei Zhang, Liping Wang, Min Wang, Yuanyuan Liu, Yan Lu, Yunpeng Liu, Chengshi Quan

**Affiliations:** 1 The Key Laboratory of Pathobiology, Ministry of Education, Beyuthune Medical College, Jilin University, Changchun, Jilin, China; 2 Department of Pathology, The 2nd Affiliated Hospital of College of Medicine, Zhejiang University, Hangzhou, Zhejiang, China; 3 Department of Thoracic Surgery, The First Bethune Hospital of Jilin University, Jilin University, Changchun, Jilin, China

**Keywords:** Breast invasive ductal carcinomas, ASK1, Apoptosis, Tight junction

## Abstract

**Background:**

Previous studies have demonstrated that claudin-6 functions as a cancer suppressor in human MCF-7 breast cancer cells. The growth inhibitory effect could be attributed to inhibition of cell proliferation and induction of apoptosis. The purpose of the current study was to examine the involvement of apoptosis signal-regulating kinase 1 (ASK1) in the anticancer effect of claudin-6.

**Methods:**

Immunohistochemical analysis was performed to evaluate the ASK1 protein expression and the correlation between ASK1, claudin-6 and clinicopathological features in 85 samples of breast invasive ductal carcinomas (IDC). Western blotting and RT-PCR was carried out to examine the expression of ASK1 and claudin-6 in MCF-7 cell clones transfected with claudin-6.

**Results:**

Immunohistochemical analysis showed that ASK1 expression was significantly related with that of claudin-6 in breast invasive ductal carcinomas (***P*** < 0.05). In addition, a positive correlation between ASK1 and C-erb B 2 protein expression was identified (***P*** < 0.05). Western blotting and RT-PCR consistently revealed that the level of ASK1 protein and mRNA was upregulated in MCF-7 cell clones transfected with claudin-6.

**Conclusions:**

Our data suggests, for the first time, that the ASK1 signal may play a positive role in the inhibitory effect of claudin-6 in breast cancer.

**Virtual Slides:**

The virtual slide(s) for this article can be found here: http://www.diagnosticpathology.diagnomx.eu/vs/1200314318763661

## Background

Breast cancer is one of the most frequent and deadly cancers in women [1]. Emerging evidence points toward a pivotal role of tight junction (TJ) in mediating tumorigenic growth of breast cancer [2,3]. TJs are junctional complexes which mediate cell-to-cell adhesion in epithelial and endothelial cellular sheets [4], and which affect cell polarity and tight junction formation [5].

Claudins (CLDNs) constitute a family of integral membrane proteins and have been identified as prominent structural components of TJ strands [5,6]. The CLDNs which include 27 members at least [7], encode 20-27 kDa proteins with four transmembrane domains and two extracellular loops [8]. The expression of CLDNs is often different in various types of human tumor [9,10]. Many studies have demonstrated that claudins may participate in several signal transduction pathways [11-15]. For instance, inhibition of c-jun NH2-terinal kinase (JNK) and p38 mitogen-activated protein kinase (p38 MAPK) selectively modulates the expression of claudin-4, -8 and −9 to enhance TJ barrier function in mammary epithelial cells [16], And p38 MAPK activity is involved in the epithelial barrier dysfunction in which claudin-7 protein plays a major role [17]. It is well known that apoptosis signal-regulating kinase 1 (ASK1) phosphorylates and actives both p38 and JNK pathway [18]. ASK1 is a member of the MAPKKK family and functions as a promoting apoptosis gene in response to common pro-apoptosis stresses [19]. However, there is little knowledge about the relationship of ASK1 and claudins, especially claudin-6.

In our previous study, we found that claudin-6 is preferentially expressed in mammary epithelial cells and functions as a potential breast cancer suppressor gene [20], which is supported by the follow-up study of Osanai [21]. Recently, we have discovered that the low level expression of claudin-6 gene contributed to malignant progression of breast cancer [22]. A previous study has described that breast cancer tissues also expressed lower levels of ASK1 compared with normal mammary tissues [23]. Therefore, the purpose of the current study is to discover the relationship between ASK1 and claudin-6 in breast cancer and to explore the pathways involves the activation of ASK1.

## Methods

### Patients and tissue samples

The breast samples were obtained from 2006–2010 in the Jilin Oil Field General Hospital in Songyuan, Jilin province, China. A total of 85 breast invasive ductal carcinomas (IDC) aged 26 to 77 with a mean age of 51 were included in this study. The study was approved by the Ethics Committee of Jilin University. Clinicopathological features of 85 IDC samples are summarized in Table 1.

**Table 1:** Clinicopathological features and the expression of ASK1 in 85 IDC samples

**Clinicopathological features**	**Cases**	**ASK1 expression (n)**		***χ*2**	***P***
		**Positive**	**Negative**		
Age (years)				0.017	0.896
≥45	58	18	40		
<45	27	8	19		
Histological grade				0.668	0.414
I ~ II	21	8	13		
III	63	18	45		
Tumor size				0.21	0.646
≥5 cm^3^	66	21	45		
<5 cm^3^	19	5	14		
Lymph node metastasis				0.527	0.468
Positive	41	11	30		
Negative	44	15	29		
TNM stage				0.336	0.562
I,II	55	18	37		
III,IV	30	8	22		
Lesion location				2.406	0.121
Right	45	10	35		
Left	34	13	21		
ER				0.105	0.746
Positive	37	12	25		
Negative	48	14	34		
PR				0.425	0.515
Positive	38	13	25		
Negative	47	13	34		
C-erb B-2				5.747	0.017
Positive	27	13	14		
Negative	58	13	45		

Statistical analyzed by Pearson’s chi-square test.

### Cell culture

Human breast cancer cell line MCF-7 cell clones expressing an vector pcDNA3.1 (+) or claudin-6 were cultured as previously described [22].

### Quantitative RT-PCR

Total RNA was extracted from clone cells using TRIzol (Invitrogen, USA) following the manufacturer’s instructions. One microgram of total RNA was subjected to reverse transcription to synthesize cDNA using the M-MuLV reverse transcriptase (TaKaRa, Japan) at 42°C for 1 hour, and 0.5 ug cDNA was used for PCR. ASK1 and claudin-6 were amplified along with GAPDH as an endogenous control following the instructions of Premix LA Taq Kit (TaKaRa, Japan). The PCR reaction conditions and the primer sequences of ASK1, claudin-6 and GAPDH are shown in Table 2. After electrophoresis, the gel was captured and analyzed by the image system (Syngene, Cambridge, UK).

**Table 2:** Primers used for PCR

**Gene**	**Sequence of primers**		**Size of PCR(bp)**	**Denaturation temperature (°C)**	**Cycles**
ASK1	sense	5’-TTCACACAAAACGGATGTAACATT-3’	198	56	30
	antisense	5’-CCTAAACAGTTATGGTCACATTTTGG-3’			
Claudin-6	sense	5’-TTCATCGGCAACAGCATCGT-3’	345	58	35
	antisense	5’-GGTTATAGAAGTCCCGGATGA-3’			
GAPDH	sense	5’-TGTTGCCATCAATGACCCCTT-3’	202	56	25
	antisense	5’-CTCCACGACGTACTCAGCG-3’			

### Western blotting analysis

The Western blotting analyses were performed as described previously [22]. Primary antibodies included ASK1 (rabbit polyclonal antibody, 1:1000, Bioworlde Technology), claudin-6 (rabbit polyclonal antibody, 1:1000, Bioworlde Technology) and β-actin (mouse polyclonal antibody, 1:1000, Santa Cruz). Secondary antibodies for the detection: anti-rabbit IgG (1:2000, Proteintech Group) and anti-mouse IgG (1:2000, Proteintech Group).

### Immunohistochemistry

Immunohistochemical staining was carried out as described in our previouspublications [24]. The primary antibodies against ASK1 (rabbit polyclonal antibody, 1:200, Bioworlde Technology) and claudin-6 (rabbit polyclonal antibody, 1:200, Bioworlde Technology) were used. The negative controls were handled in the same way except PBS instead of primary antibody.

Positive-staining exhibits brown staining, Claudin-6 was shown on the cell membrane and/or cytoplasm in breast cancer tissues [24], and ASK1 expressed on the breast cancer cytoplasm according to the manufacturer’s Instructions of ASK1 antibody. Immunostaining was observed under light microscopy with 400× magnification, and positive cells, negative cells and total cells of five different visual fields were numbered in each specimen. Scoring was performed as follows: negative (−), <10% positive tumor cells; positive (+), ≥10% positive tumor cells.

### Statistical analyses

All computations were carried out using the software of SPSS version 19.0 for Windows (SPSS Inc, IL, USA). Chi-Square test was used to examine categorical data. Unpaired t-tests were performed to evaluated data of target mRNA and protein. The data are presented as means ± standard deviation (SD) from at least three independent experiments. ***P*** < 0.05 was considered statistically significant.

## Results

### Association of ASK1 expression with the clinicopathological features of breast invasive ductal carcinomas

**Figure 1:**
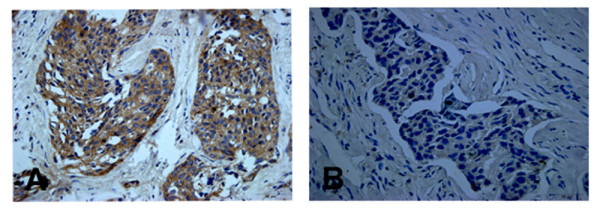
**Expression and location of ASK1 in breast invasive ductal carcinoma (IDC) (Original magnification × 400).** (**A**) ASK1 positive staining was predominant in the cytoplasm of breast carcinoma tissues. (**B**) ASK1 negative staining was seen in IDC tissues.

The clinicopathological characteristics of the patients are summarized in Table 1. In order to investigate whether ASK1 protein expression was associated with clinicopathological features of patients of breast cancer, we correlated immunohistochemical ASK1 staining results with clinicopathological features. In this study, ASK1 protein was evaluated in the cytoplasm of breast cancer (Figure 1A), and the positive expression of ASK1 protein was found in 30.59% (26/85) of breast IDCs. ASK1 protein expression had no correlation with age (***P*** = 0.896), histological grade (***P*** = 0.414), tumor size (***P*** = 0.646), lymph node metastasis (***P*** = 0.468), TNM stage (***P*** = 0.562) and lesion location (***P*** = 0.121). But interestingly, we found that ASK1 had relationship with C-erb B 2 protein expression (***P*** = 0.017).

### Correlation between the expression of ASK1 and claudin-6 in breast cancer tissues

**Figure 2:**
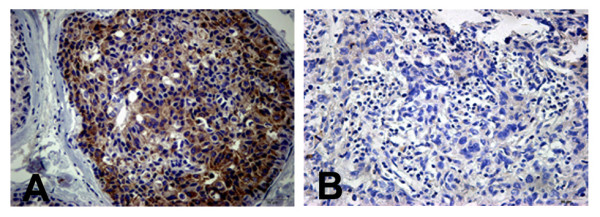
**Expression and location of claudin-6 in breast invasive ductal carcinoma (IDC) (Original magnification × 400).** (**A**) Claudin-6 was expressed in the membrane and cytoplasm of IDC tissues. (**B**) Claudin-6 was weakly expressed in IDC tissues.

We have found that the expression of claudin-6 was reduced in breast invasive ductal carcinomas [24]. The expression of claudin-6 (Figure 1A, 1B) and ASK1 (Figure 2A, 2B) was examined by immunohistochemistry, and the correlation between claudin-6 and ASK1 was analyzed by Pearson’s chi-square test. As shown in Table 3, the positive expression rate of claudin-6 was 27.09% (23/85) in IDC specimens, and cells were positive for ASK1 in 30.59% (26/85) of IDC cases. Half (13/26) of the ASK1 positive cases were positively staining for claudin-6, but only 16.95% (10/59) of ASK1 negative cases stained positively for claudin-6. Statistical analysis revealed that claudin-6 expression was positive correlated with ASK1 expression in breast invasive ductal carcinomas (***P*** = 0.0016).

**Table 3:** The correlation between the expression of claudin-6 and ASK1 in breast invasive ductal carcinomas

**ASK1 expression**	**Cases (n,%)**	**Claudin-6 expression (n,%)**		***χ*2**	***P***
		**Positive**	**Negative**		
**Positive**	26 (30.59%)	13 (50%)	13 (50%)	9.988	0.0016
**Negative**	59 (69.41%)	10 (16.95%)	49 (83.05%)		
**Case (n)**	85 (100%)	23 (27.09%)	72 (72.01%)		

Statistical analyzed by Pearson’s chi-square test.

### Correlation between the expression of claudin-6 and ASK1 in breast cancer cells

**Figure 3:**
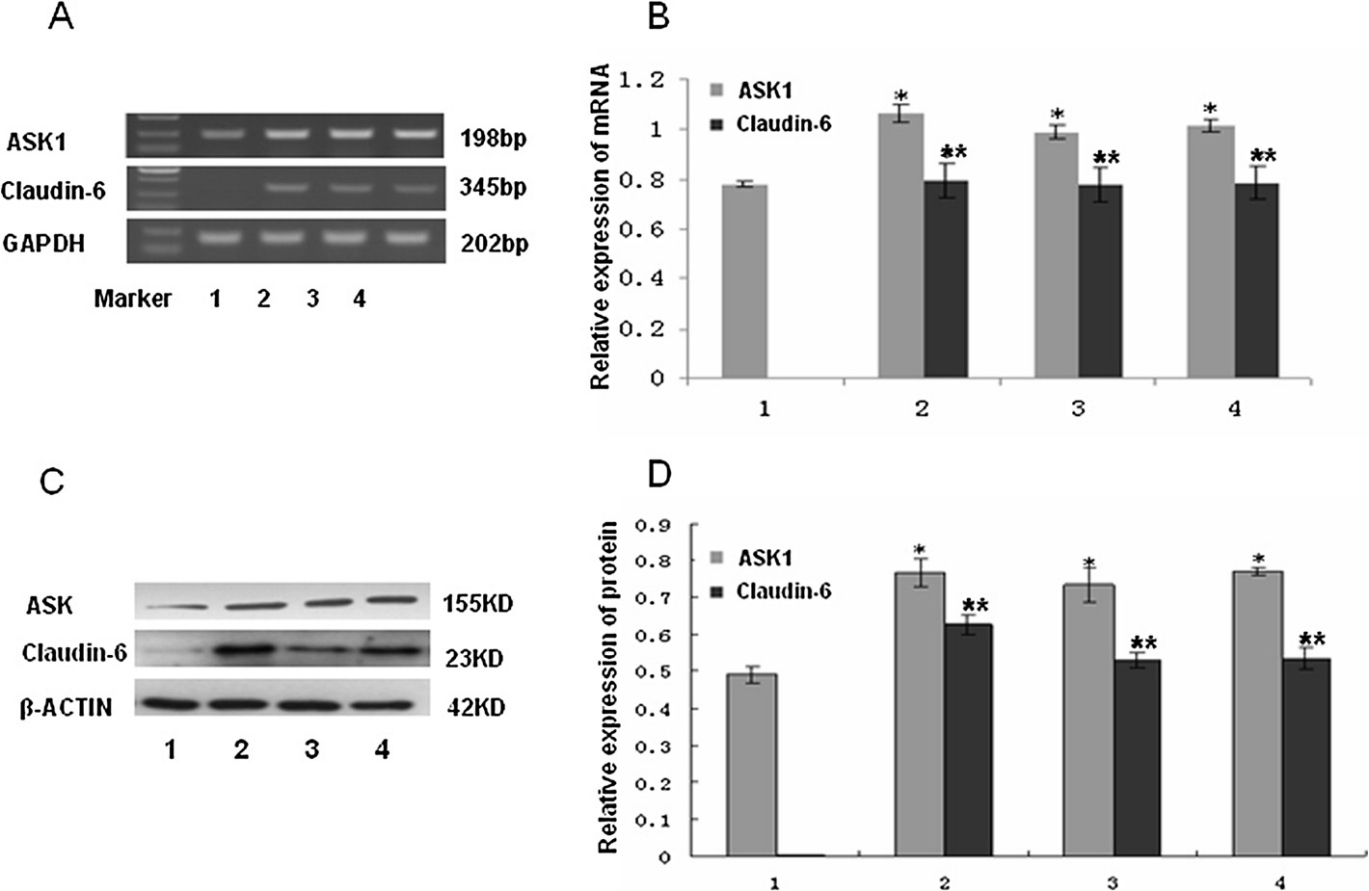
**Correlation of claudin-6 and ASK1 in breast cancer cells.** Line 1 is empty vector group; lines 2, 3 and 4 are three clone groups. (**A**) (**B**) RT-PCR assay examined the mRNA levels of claudin-6 and ASK1. (**C**) (**D**) Western blot assay detected claudin-6 and ASK1 protein expression. *,**P<0.05, vs. vector group. Results are means ± standard deviation for three independent experiments.

We found the correlation between claudin-6 and ASK1 expression in breast invasive ductal carcinomas tissues, but their relationship in breast cancer cell line was unknown. We used MCF-7 cells transfected with pcDNA3.1 (+) and three G418 resistant MCF-7 clones, which expressed claudin-6 stably. When claudin-6 was upregulated, ASK1 had a higher expression level than that in empty vector group (Figure 3A, 3C). Quantitative RT-PCR and western blot analysis showed that the level of claudin-6 mRNA and protein positively correlated with the level of ASK1 mRNA and protein (Figure 3B, 3D).

## Discussion

In the previous study, We found that the expression level of claudin-6 was lower in two human breast cancer cells (MCF-7 and BT474) and one breast cancer sample than that in normal breast tissues [20]. In addition, we also discovered the growth, migration and invasion of MCF-7 cells were inhibited by overexpression of claudin-6 [22]. One report has shown that the expression of ASK1 is lower in breast cancer tissues than that in normal tissues [23]. As we all known, ASK1 is regulated in response to various cellular stresses, including cell survival, proliferation, differentiation, and so forth. Therefore, in the current study, we attempted to elucidate the relationship between the expression of claudin-6 and ASK1 using clinicopathological features and classical prognostic factors in breast pathology, including the expression of immunohistochemical markers of prognostic significance (ER, PR, C-erb B 2) (Table 1). To the best of our knowledge, this is the first study to demonstrate an association between the protein expression of claudin-6 and ASK1 in a large series of breast invasive ductal carcinomas and the breast cancer cells.

We have previously found that the expression of claudin-6 was negatively correlated with lymphatic metastasis of breast IDCs [24], but we did not found the correlation between ASK1 expression and the lymphatic metastasis (Table 1). This may be mainly due to that the ASK1 signal pathway is not the only pathway to be regulated by claudin-6. Besides that, we found the correlation of claudin-6 and ERα (*p* = 0.033), and also discovered that ERα regulated claudin-6 in MCF-7 cells [25]. We failed to find the correlation between ASK1 and ER. And the reason high likely is the cross-talk among different signaling pathways, as we discussed in the case of failing to discover the correlation of ASK1 and lymphatic metastasis. However, we revealed the correlation between ASK1 and C-erb B 2 (Table 1). These results indicate the role of C-erb B 2 in ASK1 signal pathway. We next analyzed the relationship of C-erb B 2 and claudin-6, but we found no relationship between them (data not shown). Therefore, these data suggest that the inhibitory effect of claudin-6 in breast cancer mainly results from the regulation of ASK1.

Besides analysis of the breast cancer tissues, we also analyzed the correlation of ASK1 and claudin-6 mRNA and protein in breast cancer cell lines. We have found claudin-6 was a anti-cancer gene in claudins family [22], and the up-regulation of claudin-6 has important clinical implication, but details of the mechanism was not clear. C-jun NH2-terinal kinase (JNK) and p38 mitogen-activated protein kinase (p38 MAPK) signal pathway played a positive role in the process of claudin-4, -8 and −9 enhancing TJ barrier function in mammary epithelial cells [16]. ASK1 actives JNK and p38 pathway and induces apoptosis in various cells through mitochondria-dependent caspase activation [18,26-28]. ASK1 activation depends on its binding proteins such as TNF receptor-associated factors2/6 [29], DAXX [30], TRADD [31], RIP1 [32], and FADD [33]. And several cellular proteins, for example, thioredoxin [26], Hsp90 [34] and 14-3-3 [35] were also reported to interact with ASK1 and inhibit ASK1 activity. Here, we demonstrated that ASK1 was upregulated when claudin-6 gene was transfected into MCF-7 cells (Figure 3). Therefore, the present study indicates that ASK1 signal participates in the pro-apoptosis function of claudin-6.

## Conclusions

As a conclusion, our study suggests that the ASK1 expression is low in breast cancer, and the levels of ASK1 mRNA and protein expression are correlated with that of claudin-6. We have identified a novel mechanism responsible for the pro-apoptosis function of claudin-6, and ASK1 may become a target for breast cancer treatments. However, we still need further study to clarify the detail of this mechanism.

## Competing interests

The authors declare that they have no competing interests.

## Authors’ contributions

CQ, YG and XX carried out most of experiments, participated in the design of the study, performed the statistical analysis, and drafted the manuscript. ZL, TZ and XZ carried out part of experiments, and helped draft the manuscript. LW, MW, YL, YL and YL assisted the experiments. XX and YL helped to edit the paper. All authors have read and approved the final manuscript.
